# In-Session-Reflective-Functioning in Anorexia Nervosa: An Analysis of Psychotherapeutic Sessions of the ANTOP Study

**DOI:** 10.3389/fpsyt.2022.814441

**Published:** 2022-05-23

**Authors:** Almut Zeeck, Svenja Taubner, Thorsten C. Gablonski, Inga Lau, Stephan Zipfel, Wolfgang Herzog, Beate Wild, Hans-Christoph Friederich, Gaby Resmark, Katrin Giel, Martin Teufel, Markus Burgmer, Andreas Dinkel, Stephan Herpertz, Bernd Löwe, Sefik Tagay, Jörn von Wietersheim, Martina De Zwaan, Max Zettl, Alexander F. Meier, Armin Hartmann

**Affiliations:** ^1^Center for Mental Health, Department of Psychosomatic Medicine und Psychotherapy, Faculty of Medicine, University of Freiburg, Freiburg im Breisgau, Germany; ^2^Center for Psychosocial Medicine, Institute for Psychosocial Prevention, University Hospital, Heidelberg, Germany; ^3^Department for Psychology, Psychotherapy and Psychoanalysis, Institute for Psychology, University of Klagenfurth, Klagenfurth, Austria; ^4^Department of Psychosomatic Medicine and Psychotherapy, University Hospital Tuebingen, Tuebingen, Germany; ^5^Center for Psychosocial Medicine, Department of General Internal Medicine and Psychosomatics, Heidelberg University Hospital, Heidelberg, Germany; ^6^Department of Psychosomatic Medicine and Psychotherapy, LVR Hospital Essen, University of Duisburg-Essen, Essen, Germany; ^7^Department of Psychosomatic Medicine and Psychotherapy, LWL-Hospital Muenster, Muenster, Germany; ^8^Department of Psychosomatic Medicine and Psychotherapy, University Hospital Muenster, Muenster, Germany; ^9^Department of Psychosomatic Medicine and Psychotherapy, School of Medicine, Technical University of Munich, Munich, Germany; ^10^Department of Psychosomatic Medicine and Psychotherapy, LWL-University Clinic, Ruhr-University Bochum, Bochum, Germany; ^11^Department for Psychosomatic Medicine and Psychotherapy, University Medical Center Hamburg-Eppendorf, Hamburg, Germany; ^12^TH Köln, University of Applied Sciences, Köln, Germany; ^13^Department of Psychosomatic Medicine and Psychotherapy, Ulm University Medical Center, Ulm, Germany; ^14^Department of Psychosomatic Medicine and Psychotherapy, Hannover Medical School, Hannover, Germany

**Keywords:** eating disorder, anorexia nervosa, mentalizing, theory of mind, psychotherapy, reflective functioning, outcome

## Abstract

**Objective:**

Previous research suggests that patients with anorexia nervosa (AN) show an impaired capacity to mentalize (reflective functioning, RF). RF is discussed as a possible predictor of outcome in psychotherapeutic processes. The study aimed to explore RF in sessions of patients with AN and its association with outcome and type of treatment.

**Methods:**

A *post-hoc* data analysis of selected cases from a randomized trial on outpatient psychotherapy for AN was conducted. Transcripts from 84 sessions of 28 patients (early phase, middle phase, and end of treatment) were assessed using the In-Session-Reflective-Functioning-Scale [14 cognitive-behavior therapy, enhanced (CBT-E); 14 focal psychodynamic therapy (FPT); 16 with good, 12 with poor outcome after 1 year]. Relations between the level of RF, type of treatment, and outcome were investigated using mixed linear models. Additionally, associations with depressive symptoms, weight gain, and therapeutic alliance were explored.

**Results:**

Mean in-session RF was low. It was higher in FPT when compared to CBT-E treatments. The findings point to an association between RF increase and a positive outcome. An increase in BMI in the first half of treatment was associated with higher subsequent in-session RF. There was no association between RF and depressive symptoms or the therapeutic alliance.

**Discussion:**

Patients with AN show a low capacity to mentalize in sessions, which seems to be at least partly dependent on the degree of starvation. The results suggest a possible relationship between an increase in in-session RF and outcome, which has to be replicated by further studies.

## Introduction

Mentalizing is conceptualized as the ability to be aware of or imagine mental states in oneself and others [“seeing ourselves from the outside and others from the inside,” p. 5, (1)] and how these mental states relate to actions and behavior. Mentalizing (operationalized as “reflective functioning,” RF) is a capacity that is relevant for the regulation of self and intense emotions, as well as interpersonal functioning (2). The theory of mentalization, which is based on psychodynamic and attachment theory as well as developmental psychology and the cognitive concept of the “theory of mind,” was first described by Fonagy (3). It is assumed that the capacity to mentalize develops, at least to a large extent, during the first years of life, depending on the quality of the interactions between a child and its primary caregivers. Fonagy and Allison (4) further introduced the concept of “epistemic trust,” which postulates that an individual will only be open to new information coming from others if she/he considers them trustworthy and the information relevant to the self. A prerequisite for developing epistemic trust is thought to be an individual's experience of being recognized by others and perceived as a thinking and feeling human being. Hence, when transferring this to the psychotherapeutic process, Fonagy and Allison (4) suggest that there is a close link between a mentalizing therapist, the development of epistemic trust in the patient and her/his mentalizing, and the patient's openness for new experiences and change.

In anorexia nervosa (AN), difficulties in the regulation of emotions and self-esteem, as well as interpersonal problems, are core problem areas that are linked to psychopathology (5–10), e.g., restrictive eating and weight loss or binge-purging behavior, as well as the maintenance of the disorder [see also the cognitive-interpersonal model of anorexia nervosa (11, 12)]. In terms of psychodynamics in relationships, there is a predominance of insecure attachment patterns and a strong wish for autonomy, while feeling needy and dependent on important others (13, 14). Additionally, most patients are highly ambivalent regarding change, tend to avoid new experiences, and show reduced cognitive flexibility (15). Previous findings also point to a reduced capacity to mentalize (16–19), which is supported by clinical contributions and qualitative empirical work (20, 21). Additionally, in one study, a relationship between a higher capacity to mentalize and better outcomes was found (22). Difficulties in self-related information processing and perspective taking were also supported by findings from neuroimaging studies [see e.g., (23)]. While physical self-perception seems to normalize in recovered patients with AN, tasks on social self-perception still showed differences in comparison to healthy individuals. In sum, a low capacity to mentalize might be a predisposing or maintaining factor concerning the abovementioned difficulties. However, the number of studies assessing mentalizing in AN is small [for a recent review, see (24)]. More studies can be found that examine a related concept, the theory of mind (ToM). ToM is defined as the ability to represent the mental states of others and use them to understand and predict behavior. Findings so far point to difficulties in ToM as assessed in standardized tasks in patients with AN, but also to significant interpersonal variability (25–27). It is not yet clear to what extent difficulties are state-dependent (e.g., related to the degree of malnutrition, illness duration, and comorbidity) or constitute predisposing traits. However, there is one study suggesting an influence of anxiety and depressive symptoms, which among others, includes patients with AN (28).

The theory of mentalizing has been used to develop a treatment model, which is known as “mentalization-based treatment” (MBT). This therapeutic approach was first developed for the treatment of borderline personality disorders (BPD). While it is first-line therapy for BPD, there is only scarce evidence for the use of MBT in other mental disorders although MBT might be a promising approach (1). Only a few studies have so far evaluated MBT in the treatment of eating disorders (EDs) (29–32). As these studies did not include an assessment of the therapeutic process, they do not allow us to determine whether specific interventions that aimed to improve mentalizing resulted in an increase in RF and whether such an increase was associated with a better treatment outcome.

Bateman and Fonagy (33) postulated that not only MBT but also other treatments foster mentalizing. They suggested that mentalizing (“reflective functioning”, RF) might be considered a common factor in a range of effective psychotherapeutic approaches (1, 4, 33). However, there are mixed findings concerning the role of RF as a moderator or mediator of the therapeutic process so far. First, it might be assumed that patients with high RF will be able to use psychotherapy more productively and that higher baseline RF will be associated with a better therapy outcome (RF as a moderator). This assumption was supported by some studies (34–37), but not by others (38, 39). Second, RF could be assumed a mediator of change: an improvement in RF might enable a better understanding of one's own affects and the behavior of others, thus reducing interpersonal problems or difficulties in affect regulation. Overall, improvement in RF was most consistently found in psychodynamic treatments (35, 40–43). This might be due to a focus on “insight” and reflecting on emotional processes in relationships. However, empirical evidence on the relation between RF change and outcome is sparse (44). As far as we know, there is only one study on patients with an ED showing that an increase in RF predicted symptom change in psychoanalytic psychotherapy for BN (35). Finally, it could be assumed that a higher capacity of a patient to mentalize might reduce the risk of misunderstandings in the therapeutic relationship through a better understanding of his/her own emotional reactions and the ability to reflect on the mental state of the therapist. Thus, higher RF might be associated with better therapeutic alliances, which was supported by empirical findings (34–36, 39).

Notably, the capacity to mentalize can be good or poor, but mentalizing in a specific situation is also context-dependent and has been shown to fluctuate within and between sessions in psychotherapeutic processes (41, 45). Psychotherapy sessions will differ depending on attachment-related arousal and content, and a therapist might or might not stimulate RF. Therefore, it is important to distinguish between a general capacity to mentalize and mentalizing in a psychotherapy session, although both will be interdependent. In this sense, a higher general capacity to mentalize, as measured with the Reflective-Functioning Scale applied to the adult attachment interview (AAI), was found to be associated with higher RF in psychotherapy sessions (41, 46). In sum, more research is needed to examine the relevance of mentalizing/RF in psychotherapeutic processes of different mental disorders, including EDs.

In this study, we aimed to explore for the first time the level and relevance of RF in the psychotherapeutic processes of patients with AN. The background is the question of whether a psychotherapeutic approach that takes into account the capacity to mentalize and fosters mentalizing might be helpful in the treatment of AN, in terms of outcome and terms of the quality of the therapeutic alliance. Additionally, the study aimed to explore factors that may affect RF like the degree of underweight and depressive symptoms. We used cases from, the Anorexia Nervosa Treatment of Outpatients (ANTOP) study, a randomized controlled trial comparing cognitive-behavioral (CBT-E) and focal psychodynamic (FPT) outpatient treatment for AN with an optimized treatment as usual condition (TAU-O) (47, 48). Transcripts of session audiotapes were analyzed, which were available for the CBT-E and the FPT condition only. From each treatment type, 14 cases were selected (*N*_cases_ = 28) for an in-depth session analysis, with one session each from the beginning, middle, and end of treatment (*N*_sessions_ = 84). We selected cases with either good or poor outcomes after 1 year (omitting partial remission) to better identify effects through high contrast.

The study was partly guided by the reviewed state of research and partly exploratory and hypothesis-generating (49). It had the following aims: (1) to assess the capacity of patients with AN to mentalize as shown in psychotherapy sessions (in-session-RF); (2) to assess whether there is an increase in in-session-RF over the course of treatment; (3) to assess whether an increase in RF is associated with a better outcome; (4) to assess whether RF is associated with the type of treatment; and (5) to explore whether there was an impact of weight gain on in-session RF or an association of RF with symptoms of depression and therapeutic alliance.

Based on previous findings on a reduced capacity to mentalize in AN, it was assumed that patients will show a low level of in-session-RF. We expected in-session RF to be higher in FPT compared to CBT-E sessions. It was further hypothesized that an increase in in-session RF will be associated with a better outcome, at least in focal psychodynamic treatments (FPT).

## Materials and Methods

### Recent Study

#### Design

Transcripts of audiotaped sessions from the ANTOP trial (47) were analyzed in this study. Audiotapes were available for the CBT-E and FPT conditions (160 out of 242 randomized patients). Treatments/cases for this study were selected according to the following criteria: (1) the patients completed the treatment (at least two-thirds of the 40 sessions: ≥26 sessions; *N* = 118); (2) there were audiotapes of sufficient quality from three phases of treatment (beginning, middle phase, and end); (3) patient and therapist both agreed that the audiotapes can be used for research; (4) half of the treatments should be CBT and half FPT; and (5) to contrast cases with a good and poor outcome at the 1-year follow-up (*N*_available_ = 123 of 160), all partially remitted cases were excluded (~60% in both conditions). Thus, it was aimed to realize (*post-hoc*) a balanced 2 by 2 “design” [(CBT-E | FPT) × (complete remission |still ill)]. Finally, 28 treatments could be included in the study: 14 CBT and 14 FPT treatments, 16 with a good outcome, and 12 with a poor outcome.

Sessions were coded for in-session-RF. Additionally, data from the following main measurement time points of the ANTOP study was used: baseline, end of treatment (sample description, weight, and depression scores), and 12 months after the end of treatment (overall outcome).

For more details on the study design and measures of the ANTOP study, see below (47, 48).

#### Selection of Sessions

From each case, we selected one session each from three treatment phases: sessions 1–16 represent the early treatment phase (T1); sessions 17–32 represent the middle phase of treatment (T2); and 33–40 represent the end of treatment (final phase, T3). The following sessions were drawn from the audiotape repository: early phase: session no. 6; middle phase: session no. 22; and end of treatment: session no. 36.

The rationale behind this selection was two-fold: First, a psychotherapeutic process changes over time in terms of goals and focus. It can be divided into three phases: There is an initial phase that focuses on establishing a trustful therapeutic alliance, identifying problem areas, and starting change (weight gain); a main working phase that focuses on core problem areas; and a final phase that focuses on ending treatment and preventing relapse. The FPT and CBT-E manuals started with an initial session frequency of two sessions per week (sessions 1–16), switching to a lower frequency of one session per week (sessions 17–32), and finally a frequency of one session every second week (sessions 33–40). We used this structure to define the three phases of treatment. The measurement schedule with three sessions per case also reflects it. Further criteria for selecting one session of the respective phase were as follows: It should not be the first or last session of treatment due to their very specific content (first contact; parting). Therefore, we determined a session number in the middle of a phase in which there were as many video recordings in the sample as possible. If a session for a single case was not available, the next session was chosen (e.g., session 7 instead of session 6).

#### Transcripts

Audiotapes were transcribed anonymously based on the rules for transcription by Mergenthaler (50). The sessions were divided into sequences of 3 min, so a typical session (~50 min) entailed ~17 text sequences.

#### In-Session Rating of Reflective Functioning (RF)

Usually, transcripts of AAIs serve as the basis for an assessment of RF using the RF scale (51). The RF scale has shown good interrater reliability as well as discriminant and convergent validity (52). It was slightly adapted to be used for the assessment of RF in psychotherapy sessions (53). The RF ratings range from −1 to 9 (−1 = antireflective/rejection of mentalizing/RF; 0 = no RF; 3 = unsecure, low RF; 5 = usual RF or “normal” capacity to reflect on mental states; 9 = exceptionally reflective).

#### Rater Training, Reliability

Prior to the study, raters completed training on coding of RF on the AAI using the RF scale. Raters were five experienced clinicians (5–30 years of clinical experience, psychodynamic background, and additionally trained in MBT: ST, TG, IL, AH, and AZ). Raters got a 2-day training for the RF scale by ST/Heidelberg, who herself was trained in London. First, reliability was tested using a gold standard rating on 10 AAIs. Intraclass correlation coefficients (ICC) (54) showed good to excellent interrater agreement, ICC_AAI_ = 0.73–0.93. Regarding the interrater reliability of in-session RF, all raters coded a set of 11 therapy sequences from the ANTOP study additionally. We then computed a single ICC for coding in-session RF agreement among all six raters, which indicated good interrater reliability (ICC_inSessionRF_ =.81). Finally, each of the selected sessions was rated by one of the raters.

#### Rating of Transcripts

Transcripts were rated using an adapted version of the manualized in-session Reflective Functioning Scale (46, 53). For in-session RF, 3-min-sequences were coded. When calculating a mean in-session RF score, we omitted three ratings with the lowest values as each session contains parts that only serve to clarify facts or provide information (e.g., making appointments).

### Description of the ANTOP Study

#### Design

In the ANTOP study, patients with AN or subsyndromal AN according to DSM-IV were recruited at 10 university centers (47). The patients were randomized to three treatment conditions: cognitive-behavior therapy, enhanced (CBT-E; E = enhanced: CBT for EDs as developed by Fairburn, oriented on a transdiagnostic approach), focal psychodynamic therapy (FPT), and optimized treatment as usual (TAU-O; O = optimized: stands for treatment as usual as conducted by experienced outpatient psychotherapists and additional medical monitoring and support by a family physician). Patients in the CBT-E and FPT groups received a maximum of 40 psychotherapy sessions over a period of 10 months. There was a significant BMI (kg/m^2^) increase at the end of treatment in all groups, with no difference between treatment arms. The same was true for the 12-month follow-up.

#### Interventions

In FPT, interventions aimed to work on psychodynamically relevant foci, which were identified at the beginning of the treatment (55). Treatment foci entailed dysfunctional patterns in relationships as one main focus and additional foci, either conflictual themes (for example, a conflict between dependency and individuation) or impairments in personality functioning (e.g., in emotion recognition and regulation or regulation of self-worth). In FPT, the therapist was more active compared to usual psychodynamic treatments and repetitively addressed the function of disordered eating behavior and weight in relation to the abovementioned foci.

In CBT-E, the main aims were to normalize weight and eating patterns and to change dysfunctional attitudes and cognitions through the enhancement of self-efficacy and self-monitoring (56). CBT-E comprised nine possible modules (e.g., motivation, normalization of eating patterns, conveying a model of the ED, changing dysfunctional cognitions, affect regulation, social skills, body image, self-esteem and resources, and relapse prevention), of which five were obligatory. Sessions were more structured compared to FPT sessions; the topic of a session was mutually decided and worked on; the session was summarized, and finally, worksheets, as well as advice for additional practice, were given.

#### Measures

The original measures of the ANTOP study included structured interviews as well as questionnaires (self-reported) for an assessment of eating and general psychopathology. The BMI (kg/m^2^) was measured objectively at every time point. An assessment of the therapeutic process included an assessment of the therapeutic alliance. For a more detailed description of the measures, including psychometric properties, see (47, 48).

##### Structured Interviews

Assessments by trained psychologists included interviews for the assessment of the ED [Structured Interview for Anorexic and Bulimic Syndromes, SIAB-EX (57, 58)], a SCID-I interview for the assessment of comorbid conditions (59, 60), and a PSR rating [Psychiatric Status Rating; (61)] for an assessment of the overall severity of the ED.

##### Self-Report Measures

For an assessment of eating pathology, the Eating Disorder Inventory [EDI-2; (62, 63); total score: Cronbach's α = 0.95] was used. The Patient Health Questionnaire [PHQ; (64)] was administered to evaluate general psychopathology, and the PHQ-depression score (PHQ-9) was used to assess symptoms of depression in this study (Cronbach's α = 0.88).

##### Process Measures (Self-Report)

The Helping Alliance Questionnaire was administered monthly for an assessment of the quality of the therapeutic relationship between both patient and therapist [HAQ; (65), German version: (66)]. The German version measures two correlated, but theoretically distinct constructs. The first factor measures the quality of the therapeutic relationship (Cronbach's α = 0.89), and the second factor expects or perceives positive change (Cronbach's α = 0.85). We report the results for the relationship factor (patient version) since it is a measure of trust and cooperativeness not confounded with accumulating awareness of therapy outcome in later phases of treatment.

##### Definition of Outcome

In the ANTOP study, BMI at the end of treatment and 12-month follow-up was the primary outcome criterion. For this study, we did not use the BMI but a secondary and more comprehensive outcome criterion: Remission status 1 year after treatment. For an assessment, the psychiatric status rating (PSR) was combined with the BMI: Full recovery was defined as a BMI of >17.5 kg/m^2^ and a PSR score of 1 or 2; full syndrome AN (being still ill or symptomatic) was defined as having a PSR score of 5 or 6 and a BMI of ≤ 17.5 kg/m^2^. All other individuals were classified as partially recovered.

### Statistical Analyses

For exploratory data analysis, we used methods of distribution and contingency visualization (e.g., density curves, box plots, or mosaic plots). For the description of sessions, patients, treatments, or subgroups, we computed means, standard deviations, frequencies, and correlation coefficients.

Considering the hierarchical data structure, we used mixed linear models for multivariate analyses. For two reasons, we could not consider the nesting of cases in therapists and therapists in sites: we did not have informed consent for the identification of therapists, and the sample was too small to reliably estimate therapist or site effects. The mixed models were computed with R (V4.0.4) and the package lme4 (1.1–27.1).

We did not adjust the alpha level for multiple testing because the type-I error is less relevant than the type-II error in exploratory investigations.

## Results

### Sample

For a description of the patient sample, see Table 1. Since the chosen procedure of case selection may bias the ANTOP study's randomization, we computed difference tests between the treatment conditions. There were no statistically significant differences between FPT and CBT-E patients regarding age, BMI at baseline, and further demographic and clinical variables.

**Table 1:** Sample characteristics at baseline.

	**Subgroups**		
	**CBT-E**	**FPT**	**Total**
	**(*n* = 14)**	**(*n* = 14)**	**(*n* = 28)**
**Demographic characteristics**	*M* (*SD*); *n* (%)	*M* (*SD*); *n* (%)	*M* (*SD*); *N* (%)
Gender (female)	14 (100%)	14 (100%)	28 (100%)
Age (years) at intake	28.34 (10.41)	27.81 (8.41)	28.07 (9.29)
Marital status
Single, never married	11 (78.6%)	12 (85.7%)	23 (82.1%)
Married, or living as such	2 (14.3%)	2 (14.3%)	4 (14.3%)
Separated or divorced	1 (7.1%)	0 (0%)	1 (3.6%)
**Clinical characteristics**
BMI [kg/m^2^]	16.64 (1.11)	16.36 (0.96)	16.50 (1.02)
AN-subtypes
Binge-Purge	3 (21.4%)	6 (42.9%)	9 (32.1%)
Restrictive	11 (78.6%)	8 (57.1%)	19 (67.9%)
Duration of illness
≤ 6 years	8 (57.1%)	11 (78.6%)	19 (67.9%)
> 6 years	6 (42.9%)	3 (21.4%)	9 (32.1%)
Co-morbid disorders (DSM IV)
Affective disorder	2 (14.3%)	1 (7.1%)	3 (10.7%)
Anxiety disorder	4 (28.6%)	3 (21.4%)	7 (25.0%)
Somatoform disorder	2 (14.3%)	0 (0%)	2 (7.1%)
Mean total EDI-2-Score	258.23 (55.90)	257.56 (53.38)	257.92 (53.64)

*CBT-E, enhanced cognitive-behavior therapy; FPT, focal psychodynamic therapy; n, sample size; BMI, body mass index; AN, anorexia nervosa; EDI-2, eating disorder inventory-2*.

The patients included in this study were comparable to the 160 patients receiving FPT and CBT in the ANTOP study in terms of BMI, duration of illness, mean total EDI-2 score, and marital status (*n* = 160; mean age = 27.7 years, duration of illness > 6 years = 39%, mean total EDI-2 score = 263.5, and marital status single/never married = 82%). The percentage of the AN binge-purging subtype was somewhat lower (32.1 vs. 45.5% in the whole sample) as well as a comorbid affective disorder (comorbid affective disorder: 10.7 vs. 24.5%; comorbid anxiety disorder: 25.0%).

### In-Session RF, Descriptively

Table 2 summarizes the RF trajectories of all 28 cases by treatment arm and outcome. Descriptively, in the initial treatment phase, the mean RF was 3.06 (*SD* = 0.66). In the middle phase, the mean RF was 2.93 (*SD* = 0.93), and in the final phase of treatment, it was *M* = 2.91 (*SD* = 1.00). Throughout the sessions of all treatment phases, the mean RF was rather low (below a rating of 4).

**Table 2:** In-session RF over time by treatment arm (outcome).

**Arm (outcome)**	***n***	**T_**1**_ *M (SD)***	**T_**2**_ *M (SD)***	**T_**3**_ *M (SD)***
CBT	14	2.74 (0.58)	2.37 (0.51)	2.71 (1.05)
FPT	14	3.38 (0.58)	3.47 (0.94)	2.96 (1.05)
(–)	12	3.18 (0.84)	2.85 (0.97)	2.51 (1.09)
(+)	16	2.96 (0.47)	2.97 (0.92)	3.08 (0.96)
CBT(-)	7	2.73 (0.71)	2.31 (0.36)	2.38 (1.01)
CBT(+)	7	2.74 (0.47)	2.44 (0.65)	3.05 (1.07)
FPT(–)	5	3.82 (0.59)	3.61 (1.09)	2.71 (1.29)
FPT(+)	9	3.13 (0.43)	3.40 (0.92)	3.11 (0.95)

*RF, reflective functioning; T_1_, early phase; T_2_, middle phase; T_3_, end of treatment; CBT, enhanced cognitive-behavior rherapy; FPT, focal psychodynamic therapy; M, mean; SD, standard deviation*.

As RF fluctuates within a session, we additionally calculated the mean of the three highest ratings of all sessions to find out which level of RF patients achieved as a maximum: The mean of the three highest RF ratings in the initial phase was 4.1 (*SD* = 0.89), 4.0 (*SD* = 1.06) in the middle phase, and 4.0 (*SD* = 1.27) in the final phase. For examples of non-, low-, and normal mentalizing in sessions, see Supplementary Appendix 1.

The descriptive evaluation suggests a difference between RF levels of FPT and CBT-E, with higher levels in the FPT condition, but no salient pattern of change. Significance tests will be presented with the mixed models (see Table 3).

**Table 3:** Mixed models.

**Model 1 Unconditional Model: Random effects**
**Random effects:**
**Groups**	**Name**	***Var***	***SD***	***Corr***	
CaseID	Intercept	0.4018	0.6339		
	Phase_123	0.1561	0.3951	−0.61	
Residual		0.2759	0.5253		
**Fixed effects:**	***Estimate***	***SE***	***df***	***t-Value***	*P **<***
Intercept	3.164	0.193	27.0001	16.37	0.0001
Phase_123	−0.1109	0.103	27.0001	−1.08	0.2890
LME4 formula: RF ~ Phase_123 + (Phase_123 | CaseID)
**Model 2a Full Model of Fixed Effects: Effects of treatment arm and outcome**
**Fixed effects:**	***Estimate***	***SE***	***df***	***t-Value***	*P **<***
Intercept	3.039	0.266	49.9114	11.41	0.0000
Phase_123	−0.231	0.168	27.6722	−1.38	0.1789
Arm[FPT]	1.166	0.309	49.9114	3.78	0.0004
Outcome[+]	−0.802	0.312	49.9114	−2.57	0.0132
Outcome[+]:Phase_123^a^	0.432	0.196	27.6722	2.20	0.0361
Arm[FPT]:Phase_123^a^	−0.254	0.194	27.6722	−1.31	0.2023
LME4 formula:
RF ~Phase_123 + Arm + Outcome + Outcome*Phase_123 + Arm*Phase_123 + (Phase_123 | CaseID)					
^a^Estimates of interaction effects of Group(outcome or arm) with time (phase_123) = Difference of slopes					
**Model 2b: Reduced Model of Fixed Effects** ^**b**^
**Fixed effects:**	***Estimate***	***SE***	***df***	***t-Value***	*P **<***
Intercept	3.168	0.249	51.4897	12.751	0.0000
Phase_123	−0.337	0.149	28.1903	−2.267	0.0313
Outcome[+]	−0.757	0.312	44.8619	−2.428	0.0193
Arm[FPT]	0.856	0.197	29.6488	4.354	0.0001
Outcome[+]:Phase_123	0.395	0.197	28.1903	2.011	0.0540
^b^ Omitting the interaction effect of Phase_123 and outcome (not significant in Model 2a)					
LME4 formula: RF ~Phase_123 + Arm + Outcome + Outcome*Phase_123 + (Phase_123 | CaseID)					

*R-squares: Fixed effects only R${}_{\text{marginal}}^{2}$ = 0.243; Fixed and random effects R${}_{\text{conditional}}^{2}$ = 0.700*.

### In-Session-RF and Outcome

**Figure 1:**
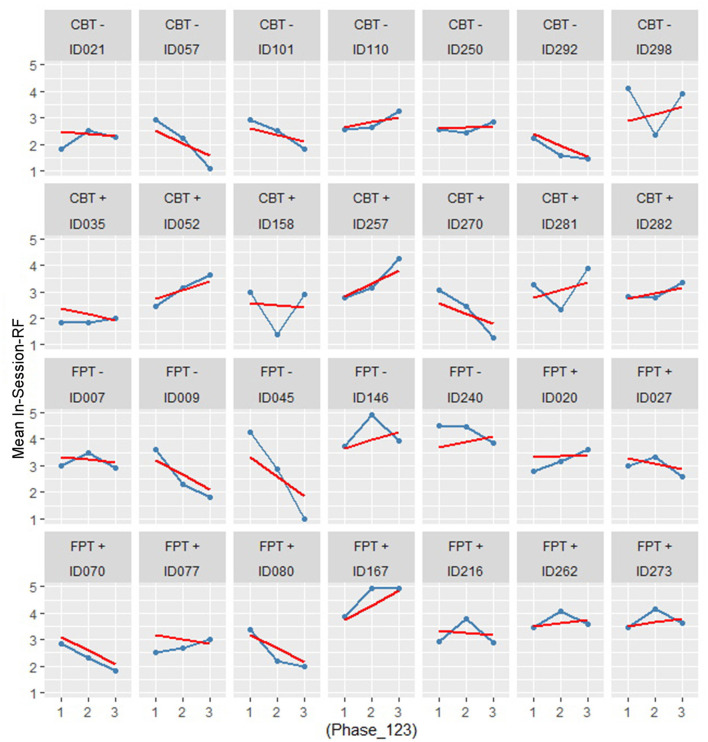
RF casewise over treatment physe by treatment arm and outcome.

Figure 1 shows the RF trajectories of all cases, with their respective treatment arm and outcome status. Linear regression lines visualizing the random effects of the final mixed model (see below) are also shown.

To test if treatment or outcome was related to RF, we set up a series of mixed models. The unconditional model comprises the treatment phase (time, coded “1” to “3”) and the session means of RF as a dependent variable nested in patients (level-1 model). The treatment arm, outcome category, and their interaction term were added stepwise as level-2 fixed effects. Table 3 summarizes the results.

The variances of the unconditional model produce a large ICC = 0.593. This high proportion of within-case variance justifies the computation of a hierarchical linear model. The estimated mean RF over all cases and time points is RF = 3.164, which is significantly different from zero. The fixed effect slope or average trend is −0.11 RF points at T2 and T3 each (not significant). This result suggests that there is no steady increase in RF over treatment time; on the contrary, the result shows a negative trend.

Model 2a (see Table 3) estimates the fixed effect of treatment arms and outcome classification on the intercept (*Int* = 3.039) and a fixed effect for the FPT-treatment arm of *Est* = +1.166. If a patient belongs to the good outcome category, he/she starts with −0.80 RF-points less than those with a poor outcome. The slope of the RF trajectory (−0.231) is not significant for the total sample, but there is a significant interaction with outcome and arm, meaning that patients with a good outcome compensate for the initial “handicap” with +0.432 RF points per time point. The treatment arms do not differ significantly in their mean slopes (−0.254).

To create a parsimonious model with better estimates, Model 2b (see Table 3) omits the insignificant arm-by-phase interaction. Overall, the results seem stable. The mean RF is at a low level (3.168). There is a significant reduction in RF over time for both arms (−0.337). Patients with a good outcome start with a lower RF (−0.757). FPT treatments show a higher RF level (0.856) *and there is a strong trend (P*<*0.054) of increasing RF* in patients with a good outcome (*P* <0.054).

Finally, a comparison of the two models showed that Model 2b performs significantly better than Model 2a, meaning that the included predictors add a relevant proportion of the explained variance to the model (fixed-effects *R*^2^ = 0.243; fixed and random effects *R*^2^ = 0.700, see Table 4).

**Table 4:** Comparison of models.

	***Npar***	***AIC***	***BIC***	***logLik***	***deviance***	***Chisq***	***df***	***P < ***
Model 1	5	203.1	215.25	215.25	193.1			
Model 2b	9	190.81	212.69	−86.406	172.81	19.096	3	0.0003

*Npar, number of parameters; AIC, Akaike's information criterion; BIC, Bayesian information criterion, logLik, log flikeliness*.

### Correlations of RF With Depressive Symptoms, BMI, and Alliance

The correlations of in-session RF with depression scores (PHQ-9), BMI, BMI change, and the therapeutic alliance in the whole sample (*N* = 28) are shown in Table 5. RF was not associated with depressive symptoms, the alliance, or BMI at any of the available time points. However, we found one significant time-lagged correlation of weight gain from Phase 1 to Phase 2 (BMI differences T2 – T1) with RF in Phase 3: an initial weight gain seems to be a precursor of higher RF in the termination phase.

**Table 5:** Correlations of RF with depression, alliance, and BMI.

**Correlations**	**Row time point(s)**	**RF at**		
		**T1**	**T2**	**T3**
Depression	As column	0.14	–	0.04
HAQ-Relation	As column	−0.26	−0.15	0.04
BMI	As column	−0.26	0.15	0.44
Change of BMI	T1 → T2	0.16	0.13	**0.53**
	T2 → T3	−0.18	0.03	0.05

*RF, reflective functioning; T1, early phase; T2, middle phase; T3, end of treatment; Depression, PHQ-9 score; HAQ relation, Helping Alliance Questionnaire, factor measuring the quality of the relation; Change of BMI, BMI difference T2 – T1 or T3 – T2, positive values in case of weight gain; **bolded** correlations: P <0.05, all others n.s*.

## Discussion

This study aimed to explore the level of mentalizing (reflective functioning/RF) in psychotherapy sessions of patients with AN and its relation to outcome and type of treatment. Additionally, associations with weight as well as depressive symptoms and the quality of the therapeutic alliance were explored. As far as we know, it is the first study on RF in patients with AN in an outpatient setting. The patients did receive two evidence-based, manualized psychotherapies for AN: CBT-E and FPT. A selected subsample of the original sample of the ANTOP study was included, balanced according to treatment approach and long-term outcome. CBT-E and FPT patients were comparable concerning sociodemographic characteristics, BMI at baseline, and severity of the ED.

In sum, patients with AN showed a low level of reflective functioning (RF) in sessions. On average, the level of RF did not change over the course of treatments and even showed a slight decrease. Sequences in which mentalizing took place (higher mean RF in a session) were more often found in sessions of FPT compared to CBT-E treatments. An increase in RF seems to be associated with a better outcome 1 year after the end of treatment. However, the trajectories of in-session RF varied considerably between patients. The findings further suggest that a reasonable weight might be a prerequisite for mentalizing in the patient–therapist interaction.

In-session RF ratings from the first treatment phase showed a level of about “3,” which refers to a low capacity to mentalize [Fonagy et al. (51)]. This level did not change significantly over the course of treatment. The finding of low RF scores is in line with findings from previous studies on patients with AN (16, 18, 19), although a direct comparison of RF values is difficult as the measures used for RF assessment differed. The finding is also in line with qualitative research showing an impaired symbolic capacity with a closeness of emotional and bodily experiences in patients with AN (21) as well as with research that found evidence for impairment in the ToM (67). Interestingly, a recent systematic review and meta-analysis differentiating between cognitive and affective empathy in EDs identified impairment in cognitive but not affective empathy in patients with AN in comparison to healthy controls (27). This means that patients with AN seem to be capable of sharing the feelings with others, but have difficulties understanding and interpreting them. In this meta-analysis, it was not possible to test for possible influences of BMI or illness duration due to a lack of studies reporting on these factors (27). However, especially BMI might be an important factor (68). At this point, it has to be noted that RF in this study was assessed as in-session RF during a psychotherapeutic process. The RF measurement was not based on an interview with standardized questions that demand mentalizing like the AAI or measured with tests as used for assessing ToM. Therefore, RF in this study can be considered a “snapshot” assessing a state in a specific situation, which refers primarily to the potential of a dyad in a “here-and-now” situation (patient–therapist interaction, embedded in the techniques of a “school” of therapy). Furthermore, it is not the explicit goal of CBT-E and FPT to promote RF, although it can be assumed that this happens implicitly, e.g., when reflecting on dysfunctional cognitions or dysfunctional patterns in relationships. Unfortunately, in the ANTOP study, there were no AAIs available from baseline, which would have allowed an application of the RF scale with a more general assessment of the capacity to mentalize. As inside a session, there are many sequences with non-mentalizing and some patients probably have a better capacity to mentalize as their mean RF in sessions show, we additionally calculated the means of the three highest RF ratings of the sessions (RF_3max_: *M* = 4.02; *SD* = 1.07; *N* = 84), showing that the mean in-session RF underestimates the patients' mentalizing capacities. However, this is still lower than the average score of “5” in a healthy population (44).

We found a significant difference in the level of in-session RF between FPT and CBT-E treatments. Since there was no assessment of RF before the beginning of therapy, we cannot rule out that CBT-E and FPT patients differed in their baseline RF. However, we think it is more likely that the RF difference reflects differences in both treatment approaches and already in the first sessions. That is, FPT might induce mentalizing (RF) more than CBT-E, although both treatments to some extent will entail interventions that promote mentalizing. Higher RF levels were shown in psychodynamically oriented treatments before (35, 40, 42, 43). The difference might be explained by the interventions used, as FPT works with interventions that focus on conflictual themes and problems in relationships and will, according to the procedures in psychodynamic approaches entail several interventions that directly induce a reflection on the self and others' states of mind (55, 69). CBT-E also focuses on these aforementioned themes, but probably to a lesser extent, and by using another kind of intervention (69); furthermore, in CBT-E, there is time reserved in sessions to go through homework and practical advice, which both can be assumed to prevent mentalizing. Overall, the finding of differences in in-session RF levels across all treatment phases suggests that in-session RF, although depending on the general ability of a patient to mentalize, might be dependent on the extent to which mentalizing is fostered inside a session. However, many other factors could have influenced mentalizing in a session (e.g., the content of the session and the mood of the patient or the therapist). There is only one study so far on patients with BPD that showed a relationship between interventions that stimulate RF (adherence to MBT principles) and the level of RF in a session (70). It was also found that the general pretreatment RF ratings were higher than the RF levels found in single psychotherapy sessions. The same might be true for AN; the circumstances for sequences in which RF can be shown might be limited and the average capacity of patients to mentalize underestimated.

In this study, there was no significant increase in mean in-session RF over the course of treatments; on the contrary, on average there was even a slight decrease. This could be related to the treatments, patient factors, or the context-dependency of in-session RF. However, statistical models suggest an association between an increase in in-session and a better outcome after 1 year. The latter finding could not be explained by the type of treatment (CBT-E or FPT), which both do not seem to alter in-session RF overall. The finding has to be interpreted with caution, as the number of cases is small. If the result can be replicated in future investigations, then RF may be a mediator of treatment success in AN in general and should be focused on in psychotherapeutic treatments more explicitly. Surprisingly and in contrast to previous findings [see e.g., (35, 39)], we could not show that higher in-session RF was associated with a better patient-rated therapeutic alliance. Thus, patients with a higher capacity to mentalize in sessions did not do better with establishing positive therapeutic alliances. Again, this might be due to the fact that we measured in-session RF and not the general capacity to mentalize. It could also be due to specific expectations, wishes, and fears of patients with AN that often relate to concrete topics such as eating and weight, and are often not mentalized. Future studies have to show whether a treatment like MBT, which takes the capacity of a patient to mentalize into account, would be associated with a higher level of patient-rated therapeutic alliances compared to other approaches. MBT includes a specific therapeutic stance of curiosity, not-knowing, and mutual understanding, as well as validating interventions in situations of non-mentalizing of a patient. This therapeutic stance might be helpful for patients with AN with their strong wish for concrete help but also for control and autonomy and a tendency to submit and please important others, e.g., their therapist.

In our exploratory analyses, we found that a change in BMI in the first half of treatment was associated with higher in-session RF in the last treatment phase. There is evidence pointing to the suppression of emotions in stages of low weight (58). Furthermore, in a situation of malnutrition, concrete thoughts about food (in a non-mentalized mode) might precede a reflection on mental processes in oneself and others to ensure survival. However, another explanation is also possible: after patients succeeded in gaining some weight, the therapists shifted to other topics and interventions, which were associated with more mentalizing. For tailoring the therapeutic process, this could mean that at the beginning of treatment, patients should be supported to gain weight (for example, by psychoeducation and work on eating behavior), as one cannot expect a reasonable level of RF. In the following and when patients are in a more stabilized physical condition, a focus on mentalizing might be adequate.

There are several limitations to this study. First of all, the sample size is small and the statistical power is limited. The sample size does not allow for subgroup analyses, e.g., differentiating between patients with the restrictive or binge-purging subtype of AN. The study sample and the whole group of patients receiving CBT-E or FPT in the ANTOP study were comparable in terms of age, BMI, and symptom severity, but it cannot be ruled out that the subsample differed in further characteristics, e.g., comorbid disorders or baseline RF. Drawing only three sessions per case may have missed relevant processes and better in-session RF. Furthermore, no AAI interviews were available that would have allowed an overall and structured assessment of the patients' overall capacity to mentalize.

The strengths of the study entail a sample that was well described, randomly assigned, and that received manualized evidence-based treatments. In-session RF ratings were coded by trained and reliable raters using several sequences per session.

Future studies should investigate more systematically if the stage of starvation has an impact on the capacity to mentalize in patients with AN. Large sample studies are needed to identify clinically relevant subgroups of patients, as previous studies pointed to considerable heterogeneity in related constructs like ToM in patients with AN (25). Additionally, prospective studies are warranted that examine the relationship between therapeutic interventions and in-session RF as well as RF as a possible moderator and mediator of the psychotherapeutic process.

## Data Availability Statement

The raw data supporting the conclusions of this article will be made available by the authors, on reasonable request.

## Ethics Statement

This study was approved by the ethics committee of the University of Tübingen (No 440/2006) and the local ethics committee of the University of Freiburg (No 59/07). The patients/participants provided their written informed consent to participate in this study.

## Author Contributions

AZ, AH, and ST: study conception and design. AZ, AH, ST, TG, IL, and AM: acquisition of data (recent study). SZ, WH, BW, H-CF, GR, KG, MT, MB, AD, SH, BL, ST, JW, and MZ: study coordinators, acquisition of data (ANTOP-study). AH and MZ: data analysis. AZ, AH, and ST: interpretation of data. AZ and AH: drafting of manuscript. All authors: critical revision. All authors contributed to the article and approved the submitted version.

## Funding

The ANTOP-study was funded by the BMBF (01GV0624). There was no funding of this secondary data analysis. This article processing charge was partly funded by the Baden-Wuerttemberg Ministry of Science, Research and Art and the University of Freiburg in the funding programme Open Access Publishing.

## Conflict of Interest

The authors declare that the research was conducted in the absence of any commercial or financial relationships that could be construed as a potential conflict of interest.

## Publisher's Note

All claims expressed in this article are solely those of the authors and do not necessarily represent those of their affiliated organizations, or those of the publisher, the editors and the reviewers. Any product that may be evaluated in this article, or claim that may be made by its manufacturer, is not guaranteed or endorsed by the publisher.
