# Supplementary material for: In-Session-Reflective-Functioning in Anorexia Nervosa: An Analysis of Psychotherapeutic Sessions of the ANTOP Study
Source: Front Psychiatry. 2022 May 23;13:814441. doi: 10.3389/fpsyt.2022.814441 (PMC9169151; doi:10.3389/fpsyt.2022.814441)
Supplement: Supplementary file 1 [file Data_Sheet_1.docx]

**Appendix 1**

**Examples for non-mentalizing, insecure/low mentalizing and average mentalizing in sessions**

| **RF < 3: Non-mentalizing**  **Definition: Mental states are not addressed.** |
| --- |
| **Example 1:**  **P:** I have to say I had no problems at all on vacation. Because it was a completely different vacation now, going out really late for dinner in the evening.  **T:** Mmm  **P:** We always went out to eat at 9, and that's in Italy with pizza, pasta and so on.  **T:** And did you eat more than usual?  **P:** I ate more calories and sometimes also a hot meal, which I wouldn’t have eaten before. I have to say I've also noticed that previously when I didn't eat so much or I ate really healthily, I lost half a kilo - but that's no longer the case.  **T:** Is that good?  **P:** I think that’s better than this constant up and down.  **Example 2:**  **P:** From the clothes you can see that it looks different. And with the parents we also went out to eat twice.  **T:** That was all completely normal and relaxed?  **P:** Yeah...  **T:** Did you also have the feeling that they weren’t so controlling anymore?  **P:** Yeah - especially my father was always like that, he always teased me, and my mother too. And I also notice my father dares to hug me again.  **T:** So you notice that everyone is more relaxed?  **P:** Yeah, we also went round to my aunt's house. She said: "You look good" and many people have told me that now. |
| **RF = 3: Insecure/ low mentalizing**  **Definition: Mental states are only superficially mentioned, not comprehensibly provided with an example or reproduction of stereotypes.** |
| **Example 1:**  **P:** .....I see some things from a different perspective now. I have also already changed some things at home. When my husband started to prepare everything at the breakfast table again, or sometime during dinner, I said: "You know, I can't do that now". And then he's offended, but I don't care.  **T:** What does that mean, “I can't do that now”?  **P:** He has to list everything down to the smallest detail. Every point hurts like hell.  **T:** What does he list?  **P:** From his friends, the sister in the psychiatric ward and the husband wakes up again from a coma. That's bad, that she practically loses her husband at 28, but when he then talks about it all the time in every detail....  **T:** And what do you say to him then?  **P:** I tell him that and then I break off hard as nails. I don't have to know every point in detail again.  **Example 2:**  **P:** My mother cares for an old woman in a wheelchair, who often wants the windows cleaned, so my mother goes there too.  **T:** Mmm - …and your family doesn´t have the mother Saturday lunchtimes or Friday evenings.  **P:** Yeah.  **T:** Is there ever any trouble?  **P:** Yeah, there often is. We accept what she does. But my brother can’t tolerate the old woman at all. Then I say, leave Mom to it, she needs that. We also think it's great that she does that. Only because we see that she is sometimes so exhausted, because she’s working at school the other days. She’s often the idiot who has to do something extra, because she can't say no.  **T:** Mmm - that means that she behaves so socially that you and your brother have to pay for it. How do you experience that?  **P:** I am also someone who says "yes" to everything and who would do almost anything for everyone else. |
| **RF ≥ 4: Average mentalizing**  **Definition: Reflection on mental states (own or others), that has at least one of the following criteria: knowledge of the nature of mental processes, an effort to work out the underlying mental processes of behavior, the recognition of a development perspective or a reflection on mental processes related to the therapist.** |
| **Example 1:**  **P:** The last two weeks have been really exhausting. If I want to move forward for me, **I have to say a bit more about what bothers me. But it depends on how I say it.** There's already been a question from colleagues about whether I'm so toxic because I'm malnourished.  **T:** How did that affect you?  **P:** First I had to fight back again. Yeah, it was just that I was annoyed. Some of the people there are completely dependent and don't see at all when I walk around with a huge cardboard box. Everyone sits there and watches. No one stands up and helps me out. **Somehow many of them don't see that they should help. But in such a moment I can become really unpleasant.**  **T:** Does that also have something to do with the fact that someone told you that shouldn’t be so toxic?  **P:** Mmm ...**at that moment I saw it as a personal attack. But I was also already in a pretty bad mood when I went to work**.  **Example 2:**  **P:** I have **to be honest with you, last time I felt guilty**. **In the past, I would have been secretly happy if I had lost weight.** But if it's forty-five now, of course that's good. I would have also been satisfied if it had only been forty-four. The main thing is - more**.**  **T:** But it's normal isn’t it that there are fluctuations like that.  **P:** I must confess, my mother asks about it every time - she always waits for the session with the weighing on Friday. I didn't tell her the weight last time because I had the feeling that I made a mistake, and **above all because I didn't want her to be afraid that everything was going backwards again.** |

Note: Examples had to be translated and slightly changed for anonymization, but care was taken to ensure that the content did not change. Relevant parts that show a capacity to mentalize were bolded. For more details on RF-ratings see Fonagy et al. 1998 and Talia et al. 2015.
